# How Different Is the Annual Physical Examination of Older Migrants than That of Older Nonmigrants? A Coarsened Exact Matching Study from China

**DOI:** 10.3390/healthcare10050815

**Published:** 2022-04-27

**Authors:** Wanyue Dong, Jianmin Gao, Yue Wu, Chi Shen, Ruhai Bai

**Affiliations:** 1 School of Elderly Care Services and Management, Nanjing University of Chinese Medicine, Nanjing 210023, China; wanyuedong@njucm.edu.cn (W.D.); 13772525161@163.com (Y.W.); 2 School of Public Policy and Administration, Xi’an Jiaotong University, Xi’an 710049, China; gaojm@xjtu.edu.cn (J.G.); shenchi@outlook.com (C.S.); 3 School of Public Affairs, Nanjing University of Science and Technology, Nanjing 210094, China

**Keywords:** annual physical examination, older migrants, associated factors

## Abstract

It has become a top priority to ensure equal rights for older migrants in China. This study aims to explore how different the annual physical examination of older migrants is compared to that of older nonmigrants in China by using a coarsened exact matching method, and to explore the factors affecting annual physical examination among older migrants in China. Data were drawn from the China Migrants Dynamic Survey 2015 and China Health and Retirement Longitudinal Survey 2015. The coarsened exact matching method was used to analyse the difference in the annual physical examination of older migrants and nonmigrants. A logistic regression was used to analyse the factors affecting annual physical examination among older migrants. The annual physical examination of older migrants was 35.6%, which was significantly lower than that of older nonmigrants after matching (Odds ratios = 0.91, *p* < 0.05). It was affected by education, employment, *hukou*, household economic status, health, health insurance, main source of income, type of migration, range of migration, years of migration, having health records in local community and number of local friends among older migrants in China. Older migrants adopted negative strategies in annual physical examination compared to older nonmigrants. Active strategies should be made to improve the equity of annual physical examination for older migrants in China.

## 1. Introduction

In the past 40 years of reform and opening-up, China has undergone rapid economic development accompanied by massive internal migrants. The results of China’s 1% population sample survey show that the number of migrants was 246 million in 2015 [1]. This number is still growing, with the latest census data showing that the number of the migrant population has grown to 375 million in 2020 [2]. Report on China’s Migrant Population Development 2018 shows that the percentage of working migrants aged 16–59 reached 84% in 2015, and they played an important role in increasing urban labour force participation rate and promoting sustained urban economic growth [3]. However, with the ageing of earlier migrants and the characterized family migration, the number of older migrants continues to grow. The number of elderly migrants in China increased from 5.03 million in 2000 to 13.04 million in 2015 [4]. They are mainly composed of four groups, namely migrant workers, disabled migrants, healthy retired migrants and migrants following their children [3].

The interaction between health and migration is complex and dynamic, and migration can have an impact on the physical and mental well-being of migrants [5]. Lower healthcare utilization for disease prevention such as screening services and oral health check-ups, among migrants compared to nonmigrants was found in Europe, suggesting inequitable access to preventive health services for migrants [6,7]. Such results were observed in both developed countries and developing countries in which migrants have fewer medical treatment options and uncertainty about healthcare opportunities and health outcomes. Rural–urban migrant children were less likely to be fully immunized than urban nonmigrants and the general population in China, India and Nigeria [8]. Migrant workers face difficulty accessing medical assistance or delay seeking healthcare during illnesses in Malaysia and Bangalore city [9,10]. This problem may be more common among older migrants who face physical, psychological, and economic cost barriers caused by spatial transformation, accompanied by the characteristics of increased health risks as they age, low education levels, low income, and heavy burdens of medical expenses. The usage of health services is currently limited, although these older migrants have high health needs [11,12].

Migration includes the types of international and internal migration, where international migrants are defined as “individuals who remain outside their usual country of residence for at least one year” around the world, facing more common cultural and linguistical barriers, while internal migrants are defined as “individuals move within the borders of a country, usually measured across the regional, district, or municipal boundaries, resulting in a change of usual place of residence” [13]. Due to the unique household registration system, internal migration is more common in China. Therefore, the participants are the internal migrants in this study. According to the *hukou*—China’s household registration system—the population was divided into household registered and non-household registered based on whether the actual place of residence coincides with the place of *hukou* registration. For ease of understanding, they are referred to below as nonmigrants (residents with *hukou*) and migrants, respectively. This household registration system has been reported to be a major barrier preventing internal migrants from enjoying equal rights, such as access to housing, education, social and medical services, to nonmigrants in the areas where they live [14]. Studies from China have reported on the obstacles of the healthcare-seeking behaviours among these migrants. Employed migrants had the lowest recent physician consultation rate and the lowest annual hospitalization rate, which might be related to ‘healthy worker effect’ [15], and migrants were more likely to utilize private rather than public services for general healthcare and delivery care [16]. Currently, studies mainly focus on employees or maternal and child populations, and most of them are studies on the utilization of health-care services [17]. Considering the important role of physical examination in increased chronic disease recognition and treatment, risk factor control, preventive service uptake, and improved patient-reported outcomes [18], it is necessary to analyse the annual physical examination of migrants. Besides, Guo reported a low rate of medical behaviour among older Chinese migrants [19], but whether there is a difference in annual physical examination between older migrants and nonmigrants in China remains unknown. Although researchers have analysed the specific differences between migrants and locals in seeking healthcare services when illnesses occur in a particular area [20,21], evidence of annual physical examination among older migrants still lacks support from a wider range of data.

This study aims to explore how different the annual physical examination of older migrants than that of older non-migrants in China is by using a coarsened exact matching method, and to provide some policy implications for improving older migrants’ health by exploring what factors are associated with annual physical examination of them in China.

## 2. Materials and Methods

### 2.1. Data

Data were drawn from the China Migrants Dynamic Survey 2015 (CMDS 2015), an annual large-scale national survey of migrants since 2009 conducted by the National Health Commission; and the China Health and Retirement Longitudinal Survey 2015 (CHARLS 2015), nationally representative panel data for the Chinese population 45 years of age and older maintained by the National School of Development of Peking University [22]. The CMDS conducted a special survey for older migrants only in 2015 and collected data on 13,043 older migrants (aged 60 and above) from 9242 households. The CHARLS collected data on 20,284 respondents from 11,797 households in 2015, from which we obtained 7385 registered households over 60 years old that were nonmigrants. Detailed descriptions of the databases are available on the official websites https://chinaldrk.org.cn/wjw/#/data/classify/population/topicList (accessed on 20 August 2021) and https://charls.charlsdata.com/pages/Data/2015-charls-wave4/en.html (accessed on 20 August 2021). As our study used secondary data in the public domain, approval from institutional review boards (IRBs) was not required. After handling missing values, the sample size in the study was 17,242, including 11,622 older migrants and 5620 older nonmigrants.

### 2.2. Measures

Annual physical examination was measured by the question “Have you had a physical examination in the past year?” for older migrants in CMDS 2015. For older nonmigrants in CHARLS 2015, whether they had a physical examination was based on the response to “When did you have your last physical examination?” Only respondents who reported a time within the past 12 months were defined as having physical examination in the past year.

Older migrants were defined by three conditions: (1) people aged 60 years old and above, (2) people who remained outside their usual *hukou* location for at least one month and (3) people without *hukou* for their current living place. In contrast, older nonmigrants were those aged 60 and above who lived in the location of their *hukou*.

The covariates included in the study were age (60–69,70–79,80–89, and 90 and above; an ordinal variable); gender (male and female; a nominal variable); marital status (married and others; a nominal variable); educational level (primary school and no formal schooling, middle school, and high school and above; an ordinal variable); employment (yes and no; a nominal variable); *hukou* (urban and rural; a nominal variable); household economic status (in quintiles: the poorest from 2 to 500 yuan, poorer from 500.4 to 750 yuan, medium from 750.2 to 1000 yuan, richer from 1001.6 to 1500 yuan, and the richest from 1502.2 to 60,039.7 yuan; an ordinal variable), which was measured by the per capita monthly expenditures of the family; health status (good and poor; a nominal variable) based on self-evaluated health; and health insurance (yes and no; a nominal variable).

In addition, to explore the factors associated with annual physical examination among older Chinese migrants, a set of variables was included. The main source of income is divided into self-derived (including labour income, savings, pension, living security, and rent), family members and others (self-derived, family members, and other; a nominal variable), type of migration (rural to urban, urban to urban, rural to rural, and urban to rural; a nominal variable), range of migration (interprovincial, intraprovincial but intercity, and intracity but intercounty; a nominal variable), years of migration (less than 3 years, 3–9 years, and 10 years and above; an ordinal variable), willing to stay in current location (yes, no, and unsure; a nominal variable), having health records in local community (yes, no, and unsure; a nominal variable), and the number of local friends (none, 1–4, 5–14, and 15 and above; an ordinal variable).

### 2.3. Statistical Analysis

Categorical variables were described using rates and examined by the chi-squared test. Considering potential differences between groups from two different sampling databases, coarsened exact matching (CEM) was used to reduce imbalances in the estimation of the differences in annual physical examination between older migrants and nonmigrants. CEM is a Monotonic Imbalance Constraint (MIB) class of matching method that reduces the number of potential matches for a given covariate by a set of “coarsened” potential confounders that are matched simultaneously in order to increase the number of matches achieved [23]. It is preferable to other matching procedures in terms of reducing model dependence, and estimation error [24]. It has been shown to produce good covariate balance between groups and reduce the impact of confounding in observational causal inference [25]. To control for some potentially confounding influences of pretreatment variables, the covariates on which a match is made are first stratified (divided into discrete categories), and then the exact matching algorithm is used to match the objects in each layer according to the empirical distribution of the sample. Finally, the matched research objects were retained, and each older migrant (treatment observations) was matched to an older nonmigrant who had the same age, gender, and other covariates. In the matched data, all analyses were conducted by incorporating matched weights. After matching, the balance between covariates is evaluated by the L_1_ statistic, which is based on the difference of all pretreatment covariates in the treated group and those in the control group, ranging from 0 to 1. A good match would result in a lower L_1_. Details of this monotonic imbalance-reducing matching method can be found in other literature [26].

A logistic regression model was used to analyse the factors affecting older migrants’ annual physical examination in the unmatched group. Odds ratios (ORs) and 95% confidence intervals (95% CIs) were reported in the regression results. The regression results were two-tailed with a significance level of 0.05 (*p* < 0.05). All statistical analyses were performed using STATA 14.0.

## 3. Results

### 3.1. The Characteristics of the Participants

Table 1 shows the characteristics of the sample. The sample was mostly under 70 years old (72.8%), male (51.6%), possessed primary school and no formal schooling education (66.5%), rural (72.4%), married (81.4%), employed (51.6%), low- and middle-income (64.8%), possessed good health status (82.5%), and possessed health insurance (91.7%).

There were significant differences between older migrants and nonmigrants in age, gender, education, *hukou*, employment, household economic status, and health status (all *p* < 0.05). Compared with older nonmigrants, older migrants were more concentrated as under 70 years old (75.9% vs. 66.3%, *p* < 0.001), male (52.2% vs. 50.5%, *p* = 0.036), middle school and above education (40.4% vs. 19.1%, *p* < 0.001), urban (33.0% vs. 16.3%, *p* < 0.001), unemployed (49.5% vs. 46.1%, *p* < 0.001), possessing poorer to richer household economic status (66.4% vs. 44.1%, *p* < 0.001), and possessing good health status (88.9% vs. 69.3%, *p* < 0.001). As shown in Table 1, there was no statistically significant difference between older migrants and nonmigrants in annual physical examination (35.6% vs. 35.7%, *p* = 0.90).

### 3.2. Difference in Annual Physical Examination between Older Migrants and Nonmigrants

Table 2 shows the difference in annual physical examination between older migrants and nonmigrants. A total of 4139 (35.6%) older migrants and 2007 (35.7%) older nonmigrants reported having had physical examinations. Migration was not associated with higher annual physical examination in the unadjusted analysis (OR = 0.99, 95% CI: 0.93 to 1.06). Using a logistic regression model after coarsened exact matching, 10,347 older migrants and 5341 older nonmigrants were matched, and the L_1_ decreased from 0.431 to nearly zero (6.267 × 10^−15^). We identified that older migrants had less annual physical examination than older nonmigrants (OR = 0.91, 95% CI: 0.85 to 0.98). This persisted in the multivariable logistic regression model (OR = 0.92, 95% CI: 0.86 to 0.99). The multivariate imbalance measure L_1_ statistic before and after CEM is reported in Table 3.

### 3.3. Factors Associated with Annual Physical Examination

Table 4 shows the results of the three multivariate regression models. The first model incorporated the demographic and socioeconomic characteristics of the older migrants, the second model incorporated the migration and social integration characteristics of the older migrants, and Model III included all characteristics. In Model I, middle school education was related to higher use of annual physical examination (OR = 1.196, *p* < 0.01), but it was lower for those with a high school and above education (OR = 0.789, *p* < 0.01). Higher annual physical examination was associated with urban migrants (OR = 1.274, *p* < 0.01), good self-rated health (OR = 1.392, *p* < 0.01), and health insurance (OR = 1.467, *p* < 0.01). For employed individuals (OR = 0.786, *p* < 0.01) with richer and richest household economic status (both *p* < 0.05), annual physical examination was lower. Compared with older migrants whose main source of income was themselves, those whose incomes were mainly derived from family members and others presented lower annual physical examination (both *p* < 0.05). In Model II, annual physical examination was higher among urban-urban migrants and lower among rural–rural migrants compared with rural–urban migrants (urban to urban: OR = 1.163, *p* < 0.05; rural to rural: OR = 0.750, *p* < 0.01). Older migrants with a closer migration range have a higher utilization (all *p* < 0.01), and those whose migrations have lasted 3–9 years have higher utilization (OR = 1.136, *p* < 0.01). Having health records in local community was significantly positively associated with annual physical examination (OR = 4.375, *p* < 0.01) and having more local friends (all *p* < 0.01). The results of model III were similar to those of Model I and Model II, with only one exception: there was no significant difference in annual physical examination between older migrants whose income was mainly derived from others and whose income was mainly derived from themselves.

## 4. Discussion

We conducted secondary analysis using large and nationally representative datasets to explore the differences in annual physical examination between older migrants and older nonmigrants, and to investigate the factors associated with annual physical examination among older migrants in China. We aim to show the gap in their utilization and to provide a reference for realizing the health equity of older migrants.

The study found that the annual physical examination rate of older migrants was 35.6%, which is slightly lower than the report from Guo (39.64%) [19]; and the rate of older nonmigrants was 35.7%. There was no difference in the reported annual physical examination rates between older migrants and nonmigrants. However, when confounding factors were taken into account, the results of both multivariable logistic regression and CEM suggested a lower annual physical examination rate among older migrants. Chinese researchers reported the “healthy migrant effect” and convergence of health status during China’s internal migration process, that is migrants tend to have better health than native-born residents initially, but this physical health advantage diminished significantly with their increasing length of residence [14]. Due to poor living conditions, insufficient health knowledge, and a lack of social support and social integration [27], the migrating population is susceptible to various psychological and physical health problems. In addition, migrants are often at a disadvantage as outsiders [28]. Not only are they generally disadvantaged to residents in terms of social resources and human capital, but they also face inadaptability in social culture and customs [29]. The above factors have caused a higher need for health services and more difficulties in the decision-making process of health-related issues. Due to China’s household registration system, studies have shown that access to public health services outside the place of *hukou* is often restricted [30]. In response to this problem, the social welfare reform in China has taken measures such as encouraging qualified migrants to apply for local *hukou*, but it is still questionable whether older migrants with low education meet the restrictions on settlement clauses. In addition, some policies aim to expand social welfare, such as free physical examinations for all older people in a community [31], but whether they effectively improve the annual physical examination for older migrants needs to be verified by the latest data.

The results of Model I showed that education, employment, and household economics significantly affected the use of annual physical examinations for older migrants. This is consistent with other studies that showed that socioeconomic status, such as income and education, is an important factor influencing healthcare service utilization [32]. Surprisingly, we observed that although the elderly with middle school education had higher annual physical examination than those with primary school and no formal schooling education, for those with high school and above education, annual physical examination was reduced. Other Chinese researchers provide similar findings [33]. We consider the reason for this difference to be explained by the mediating role of health literacy in the relationship between education and health [34]. The elderly with higher health literacy were significantly less likely to have risky behaviours, and more likely to undergo health examinations regularly, report good self-rated health, and access sufficient health information from multiple sources [35], but health literacy is not just the result of formal education [36]. Findings from a comparative study reported that literacy skills in Bermuda better capture health-related knowledge/skills/behaviours than formal education [37]. This study found that higher household economic status was negatively correlated with annual physical examination. The main reason is that we used expenditures to measure household economics. Compared with disease treatment, physical examination is generally considered to be cheap, which partly explains the negative association between family expenditures and physical examination. From the perspective of the main source of income of older migrants, annual physical examination was lower among those with income coming from family members and others, which can reflect that income is an important factor in the use of healthcare services in China [38]. The relationship between employment and annual physical examination showed that employed older migrants had lower utilization. This could be attributed to the lack of time for employed migrants, which is also confirmed in other Chinese studies as an important factor preventing migrant workers from choosing medical treatment when getting sick [39].

Older migrants with urban *hukou* were associated with higher annual physical examinations compared to the rural group. The potential residential gap occurred in the case of preventive care utilization [40], in health knowledge and in the utilization of health services in China [16]. This gap could be an example of how rural migrants are disadvantaged in their access to healthcare. The annual physical examination of older migrants with good health was significantly higher than that of migrants with poor health, which is closely related to self-care awareness. In addition, health insurance participation has stimulated awareness of annual physical examination among older migrants, which contrasts with previous Chinese research [40]. Although the existing social health insurance does not include preventive health services such as physical examination, tumour screening, or vision or hearing examination, it addresses the health service needs of the insured by providing medical expense compensation when they visit a doctor, thereby promoting insured older migrants to adopt active health strategies. Based on the above analysis of demographic and socioeconomic factors, we propose formulating plans to improve health literacy and to improve individuals’ positive awareness of physical examination based on health education and cultural interventions. In addition, it is also necessary to formulate health policies to protect older migrants, especially to ensure that migrant workers enjoy the right to physical examinations for employees and to promote the realization of social equity by exploring various means, such as increasing health insurance benefits and providing transfer payments to older people without fixed incomes.

In Model II, we included features of migration and social integration. Consistent with our discussion above, migrants who came from rural places were more inclined to use less annual physical examination, which was reflected in the higher utilization of urban–urban migrants than rural–urban migrants. In addition, regarding destination, for older migrants with rural *hukou*, utilization is lower in those who migrated to rural areas than for those who migrated to urban areas, suggesting that there may be differences in healthcare access between urban and rural areas in China [41]. This observation is also shown in the utilization of urban–rural migrants, although no statistical significance was found. Compared with interprovincial migrants, those with closer distances showed higher utilization. This is consistent with the results of other Chinese studies [42], as the latter are only influenced by regional segmentation with fewer sociocultural differences [16]. Older migrants with a longer duration were more likely to use annual physical examination, as migrants’ social integration improves along with their years of residence in their destinations [43] and provides them with a better understanding of local medical service facilities [44]. The utilization of migrants with health records in the local community was significantly high, indicating the importance of social support for older migrants. Consistent with other Chinese studies [45], we found that migrants with more local friends were more likely to use annual physical examinations than migrants without local friends. Local friends could help migrants rebuild relatively rich social networks [44] and provide local and useful health information [46], which are important for older migrants to use healthcare services [46,47]. In Model III, we included all features of older migrants. The OR value with the results of factors were similar to the Model I and Model II when the additional factors were included. These results have important policy implications for the healthcare system and local governments to promote social equity. Policy-makers need to consider how to formulate targeted social and health policies and establish a more complete rural health delivery network to increase accessibility. Better information on services should also be provided to migrants, thereby improving their overall healthcare access and opportunities for health services. In addition, in the current primary healthcare reform, more resources and attention need to be placed on the migrant population, especially those who lag far behind their local peers in the use of services [48], which should not only help them establish health records in the local community to ensure that they can enjoy the same health policy as the locals, but also promote social integration by giving them spiritual care and organized community activities.

There are some limitations to this study. First, the data for our study came from 2015. This is because the CMDS conducted a special survey for older migrants only in 2015. To the best of our knowledge, this is the largest and most representative survey data on older migrants in China. Other than the CMDS 2015, the CMDS does not currently release updated data on the older migrants. To be consistent with CMDS, CHARLS 2015 was used for older nonmigrants in the study. Thus, the updated large-scale survey is needed to this issue. Second, our data were sourced from two different databases, although the matching method was used to reduce the variation in the sample, it may still lead to biased results. Besides, like other cross-sectional studies, we could not determine the cause-and-effect relationship. In addition, measures of annual physical examination were self-reported and could suffer from recall bias. To capture the use of older nonmigrants, the judgement of annual physical examination used the time of their last physical examination. Some older migrants may be excluded because of uncertain reporting times, which may lead to underestimation. Moreover, due to the availability of data, some factors that may affect annual physical examination for older migrants are not included, such as family income, which was measured by family expenditures in the analysis. Other factors, including the role of the family living mode and activities of daily living, need to be further explored once the data are available.

## 5. Conclusions

The annual physical examination needs of older migrants in China have not been fully met. Older migrants are likely to be unable to enjoy the same annual physical examination as older nonmigrants. This requires the government and public departments to adopt active countermeasures to improve the fairness of the use of annual physical examination for older migrants so as to avoid leaving this vulnerable group behind.

## Figures and Tables

**Table 1:** Characteristics of older migrants and older nonmigrants.

Variables	Setting	Total (*n* = 17,242)		Older Migrants (*n* = 11,622)		Older Nonmigrants (*n* = 5620)		*p*
		*n*	%	*n*	%	*n*	%	
Age group	60–69	12,547	72.8	8819	75.9	3728	66.3	<0.001
	70–79	3821	22.2	2254	19.4	1567	27.9 *	
	80–89	818	4.7	507	4.4	311	5.5 *	
	90 and above	56	0.3	42	0.3	14	0.3	
Gender	Male	8902	51.6	6065	52.2	2837	50.5	0.036
	Female	8340	48.4	5557	47.8	2783	49.5	
Education	≤Primary school	11,470	66.5	6924	59.6	4546	80.9 *	<0.001
	Middle school	3751	21.8	2996	25.8	755	13.4 *	
	≥High school	2021	11.7	1702	14.6	319	5.7 *	
*hukou*	Rural	12,489	72.4	7786	67.0	4703	83.7	<0.001
	Urban	4753	27.6	3836	33.0	917	16.3	
Marital status	Married	14,042	81.4	9504	81.8	4538	80.7	0.10
	Others	3200	18.6	2118	18.2	1082	19.3	
Employment	Yes	8899	51.6	5871	50.5	3028	53.9	<0.001
	No	8343	48.4	5751	49.5	2592	46.1	
Household economic status (in quintiles, RMB)	1	4221	24.5	2152	18.5	2069	36.8 *	<0.001
	2	3428	19.9	2447	21.1	981	17.5 *	
	3	3525	20.4	2830	24.4	695	12.4 *	
	4	3240	18.8	2438	20.9	802	14.2 *	
	5	2828	16.4	1755	15.1	1073	19.1 *	
Health status	Poor	3015	17.5	1287	11.1	1728	30.7	<0.001
	Good	14,227	82.5	10,335	88.9	3892	69.3	
Health insurance	Yes	15,818	91.7	10,673	91.8	5145	91.5	0.52
	No	1424	8.3	949	8.2	475	8.5	
Annual physical examination	Yes	6146	35.6	4139	35.6	2007	35.7	0.90
	No	11,096	64.4	7483	64.4	3613	64.3	

* For significant difference between older migrants and older nonmigrants in each subgroup.

**Table 2:** Association of migration with physical examination.

	Annual Physical Examination	
	OR (95% CI)	*p*
Crude	0.99 (0.93–1.06)	0.900
Multivariable logistic regression	0.92 (0.86–0.99)	0.027
Logistic regression after coarsened exact matching *	0.91 (0.85–0.98)	0.009

Notes: OR means Odds Ratio, 95% CI means 95% Confidence Interval. * Analyses based on matched samples.

**Table 3:** The L_1_ statistic before and after CEM.

Variables	Before Matching (*n* = 17,242)	After Matching (*n* = 15,688)
	L1 (Mean)	L1 (Mean)
Age group	0.097 (−0.105)	3.8 × 10^−15^ (2.7 × 10^−15^)
Gender	0.017 (−0.017)	6.2 × 10^−15^ (8.1 × 10^−15^)
Education	0.213 (0.303)	5.5 × 10^−15^ (7.2 × 10^−15^)
Residential	0.167 (0.167)	6.5 × 10^−15^ (5.1 × 10^−15^)
Marital status	0.010 (−0.010)	3.7 × 10^−15^ (1.8 × 10^−15^)
Employment	0.034 (−0.034)	6.7 × 10^−15^ (5.1 × 10^−15^)
Household economic status (in quintiles, RMB)	0.223 (0.317)	5.3 × 10^−15^ (1.3 × 10^−14^)
Health status	0.197 (0.197)	4.7 × 10^−15^ (8.0 × 10^−15^)
Health insurance	0.003 (0.003)	1.9 × 10^−15^ (3.1 × 10^−15^)
Multivariate L1	0.431	6.267 × 10^−15^

Notes: the mean is labelled in parentheses and reports the difference in means.

**Table 4:** Factors affecting annual physical examination among unmatched older migrants in China (*n* = 11,622).

Variables	Setting	Model I		Model II		Model III	
		OR	95% CI	OR	95% CI	OR	95% CI
Age group (Ref. 60–69)	70–79	1.097	0.992–1.215			1.068	0.957–1.192
	80–89	1.046	0.856–1.279			1.011	0.814–1.255
	90 and above	0.963	0.485–1.910			1.043	0.501–2.168
Gender (Ref. Male)	Female	1.039	0.959–1.125			1.055	0.968–1.150
Education (Ref. ≤Primary school)	Middle school	1.196 ***	1.085–1.319			1.173 ***	1.055–1.303
	≥High school	0.789 ***	0.691–0.902			0.765 ***	0.662–0.883
Marital status (Ref. Married)	Others	0.987	0.885–1.101			1.037	0.921–1.168
Employment (Ref. No)	Yes	0.786 ***	0.724–0.853			0.847 ***	0.775–0.926
*hukou* (Ref. Rural) ^1^	Urban	1.274 ***	1.149–1.412			—	—
Household economic status (Ref. Poorest)	Poorer	0.894	0.791–1.011			0.894	0.782–1.022
	Medium	0.983	0.872–1.107			1.011	0.886–1.153
	Richer	0.850 **	0.749–0.965			0.855 **	0.743–0.985
	Richest	0.665 ***	0.575–0.769			0.730 ***	0.621–0.857
Health status (Ref. Poor)	Good	1.392 ***	1.220–1.587			1.402 ***	1.215–1.617
Health insurance (Ref. No)	Yes	1.467 ***	1.263–1.703			1.226 **	1.044–1.440
Main source of income (Ref. self-derived)	Family members	0.845 ***	0.768–0.930			0.822 ***	0.741–0.913
	Other	0.837 **	0.706–0.992			0.834	0.694–1.003
Type of migration (Ref. Rural to urban)	Urban to urban			1.163 ***	1.058–1.278	1.124 **	1.000–1.263
	Rural to rural			0.750 ***	0.668–0.842	0.742 ***	0.658–0.836
	Urban to rural			0.883	0.643–1.213	0.844	0.611–1.165
Range of migration (Ref. Inter-provincial)	Intra-provincial but inter-city			1.435 ***	1.301–1.583	1.384 ***	1.253–1.529
	Intra-city but inter-county			1.549 ***	1.397–1.719	1.465 ***	1.317–1.631
Years of migration (Ref. Less than 3 years)	3–9 years			1.136 ***	1.032–1.250	1.126 **	1.023–1.241
	10 years and above			0.975	0.870–1.094	0.957	0.851–1.076
Willing to stay in current living place (Ref. Yes)	No			0.938	0.807–1.091	0.889	0.764–1.035
	Unclear			1.032	0.919–1.160	1.006	0.895–1.131
Having health records in local community (Ref. No)	Yes			4.348 ***	3.976–4.754	4.375 ***	3.998–4.788
	Unclear			1.102	0.969–1.254	1.094	0.961–1.246
Number of local friends (Ref. None)	1–4			2.019 ***	1.713–2.380	1.932 ***	1.636–2.281
	5–14			2.553 ***	2.177–2.995	2.412 ***	2.051–2.838
	15 and above			2.899 ***	2.421–3.472	2.760 ***	2.296–3.317

Notes: Ref. means Reference, OR means Odds Ratio, 95% CI means 95% Confidence Interval, ** *p* < 0.05, *** *p* < 0.01. ^1^ *hukou* was excluded in Model III due to collinearity with the type of migration.

## Data Availability

The data in this study are available from China Migrants Dynamic Survey 2015, which were used under license in this study, and China Health and Retirement Longitudinal Survey 2015, which could apply from institution on the website of https://charls.charlsdata.com/pages/Data/2015-charls-wave4/en.html (accessed on 20 August 2021). Data of China Migrant Dynamic Monitoring Survey 2015 are available from the authors upon reasonable request and with permission of the Migrant Population Service Center of the Chinese National Health Commission on the website of http://www.chinaldrk.org.cn/wjw/#/application/index (accessed on 20 August 2021).
